# Pathologists Overseas: A volunteer-based model for building sustainable, high-quality pathology and laboratory medicine services in low- and middle-income countries

**DOI:** 10.3389/fmed.2022.977840

**Published:** 2022-08-30

**Authors:** Emily H. Glynn, Ann Marie Nelson, Merih Tesfazghi, Roa Harb, Timothy Amukele

**Affiliations:** ^1^Department of Laboratory Medicine and Pathology, University of Washington, Seattle, WA, United States; ^2^Impala Consulting, Washington, DC, United States; ^3^Department of Pathology, Rush University, Chicago, IL, United States; ^4^Department of Laboratory Medicine, Clinical Center, National Institutes of Health, Bethesda, MD, United States; ^5^ICON Laboratory Services, ICON plc, Farmingdale, NY, United States

**Keywords:** global health, health systems strengthening, low- and middle-income countries (LMICs), pathology, laboratory medicine

## Abstract

For thirty years Pathologists Overseas (PO) has worked in low- and middle-income countries (LMICs) to provide affordable, sustainable, and high-quality pathology and laboratory medicine (PALM) services through strategic partnerships and the efforts of our large volunteer network. We address low quality diagnostic services by targeting the 3 pillars of PALM quality: human resources, systems, and quality and accreditation. To improve human resource capacity, PO and our partnering organizations provide virtual continuing education to pathologists and laboratory professionals in these countries. To improve systems, we provide laboratory information system installation and implementation support. Lastly, to improve quality and help laboratories progress toward accreditation, we support an external quality assurance program for laboratories in LMICs. As a relatively small organization, PO demonstrates that a network of dedicated volunteers, in partnership with corporations and professional organizations, can initiate sustainable change in the quality of PALM services in LMICs by focusing efforts on the core components of laboratory quality.

## Introduction

Pathology and Laboratory Medicine (PALM) are required for detection, management, and prevention of disease and thus are essential components of the healthcare system. Yet, there is broad consensus that the quantity and quality of these services in many low- and middle-income countries (LMICs) are poor (1–3). There is less consensus on how to improve the state of PALM in these countries. Pathologists Overseas (PO) is a US-based, volunteer-run organization whose mission is to improve PALM where resources are limited. Herein, we describe the work we have undertaken that has been most effective in improving practice in partnering laboratories in LMICs through a framework of PALM quality.

Before we introduce the framework, we want to share that our approach is focused on strengthening health systems rather than addressing any single disease. Historically, many global health programs, including those in PALM, have taken a “vertical” approach, where funding is channeled toward specific diseases (e.g., tuberculosis, malaria), without direct investment to strengthen the foundation of health systems (4). PALM as a discipline, regardless of disease entity, requires 3 things in order to render consistently accurate, reliable, and reproducible test results and pathologic interpretations. These are infrastructure, qualified staff, and a legal and regulatory environment that supports consistent quality standards. Building this capacity takes coordinated investment that has been historically lacking in LMICs (5).

### Framework

To this end, Pathologists Overseas (PO) has over thirty years of experience working in LMICs and approaches building laboratory capacity by targeting the 3 pillars of PALM quality: *human resources, systems, and quality and accreditation*. As a small organization, our mission is to provide affordable, sustainable, and high-quality diagnostic services in LMICs through strategic partnerships and the efforts of our large volunteer network. In order to demonstrate how a volunteer-based model can be utilized to build PALM capacity, we provide a brief history of PO followed by an overview of our current efforts in the framework of human resources, systems, and quality and accreditation.

## History of pathologists overseas

Dr. Heinz Hoenecke founded PO in 1991 with the mission to affordably and sustainably improve PALM services in LMICs through the efforts of volunteer pathologists, technologists, and laboratory scientists. As PO grew, it became evident that this mission would be better served by incorporating as a non-profit organization. Despite this formality, PO remained entirely volunteer-run for the next 30 years during which time it has organized and funded major projects in Kenya, Eritrea, Nepal, Ghana, Madagascar, Bhutan, Peru and St. Lucia as well as more limited endeavors in over 20 countries (6). Overall, these efforts can be summarized as establishing new technologies, initiating comprehensive quality control programs, upgrading laboratory facilities, providing textbooks and educational materials, developing training programs, and facilitating placement of physicians from LMICs to pathology fellowships in high-income countries (HICs).

PO’s prior anatomic pathology (AP) capacity building efforts include our first initiative in Kenya where we provided histopathology services to more than 40 mission hospitals for over 5 years through the support of over 90 volunteers. In keeping with the goal of transitioning services to local leaders, PO transferred all revenue and equipment to A.I.C. Kijabe Hospital outside of Nairobi. To meet the surgical pathology need in Madagascar, PO worked with local stakeholders to establish a histopathology lab and recruit volunteers from HICs to fill in service gaps and train local physicians. Two of these local physicians ultimately took over the clinical service and laboratory operations. In Ghana, PO was invited to establish a histopathology lab and pathology training program at Komfo Anokye Teaching Hospital. Similar AP capacity building projects were undertaken in Nepal and Peru.

Starting in 1994, PO’s efforts expanded to building clinical laboratory capacity. In Eritrea, we established a country-wide Laboratory Quality Assurance System, expanded testing to include clinically necessary care (e.g., hemoglobin A1c for diabetes), established a diabetes clinic, and helped launch a medical school. These efforts were supported for over 10 years and the Laboratory Information System (LIS) we installed has been sustained for 20 years. In Bhutan, we installed LISs that are now supporting over 20 hospitals/clinics in each county.

Although each setting was unique, there were pervasive challenges such as unreliable staffing, supply chains, and quality assurance programs, which led us to focus our work in 3 major areas. The first was expanding human resource capacity through the provision of technical support, historically by deploying volunteers to laboratories. The second was building sustainable infrastructure through the installation and continued support for LISs. The third was improving quality and moving laboratories toward accreditation by enrolling and mentoring them in External Quality Assurance (EQA) programs. These historical approaches, especially the deployment of volunteers for short-term opportunities, represented our primary strategies to build capacity for most of our history. With the advent of the COVID-19 pandemic as well as an acknowledgment of the improving landscape of staff capacity in LMICs (Table 1), we have pivoted toward virtual activities with the goal of reaching a broader audience and increasing the number of volunteers we are able to engage in a cost-effective fashion. These virtual activities include continuing education programs to support this new class of pathology and medical laboratory professionals, and digital content, such as podcasts and blogs, designed to support the flourishing of the LMIC PALM community.

**TABLE 1:** Change in pathologists and pathology trainees from 2015 to 2022.

Region (Number of countries)	Countries with pathologists (*n* = 40)		Pathologists			Pathology trainees		
			
	2015	2022	2015	2022	Δ (%)	2015	2022	Δ (%)
Francophone (17)	16	17	100	155	+55 (+55)	37	128	+91 (+250)
West Africa (5)	2	5	163	148	–15 (–10*)	220	120	–100 (–45)
East Africa (9)	8	9	183	290	+107 (+58)	97	171	+74 (+76)
South Africa (9)	8	8	280	336	+56 (+20)	104	148	+44 (+42)
Total	34	39	726	929	+203 (+28)	458	567	+109 (+24)

*Totals for West Africa reflect institution-based pathologists and do not represent the number of pathologists who have moved to private laboratories.

## Three pillars of palm quality

### Human resources

The first of the three pillars of PALM quality is human resources. A significant barrier to providing and advocating for quality services has been and continues to be a lack of trained PALM professionals in many LMICs. From 2012 to 2014, the African Strategies for Advancing Pathology, a group made up primarily of pathologists from 7 African countries and their partners in the US, Europe and Australia, performed a survey of pathologists and trainees in sub-Saharan Africa (SSA) (7).

The survey demonstrated that, with the exception of South Africa, SSA countries had less than 1 pathologist per million people. In addition, there were no pathologists in 6 countries and only 18 active training programs in the 40 SSA countries surveyed (7). While there is no clear guidance from international health organizations indicating the number of pathologists per capita (anatomic and clinical) required for functional health systems, the number in resource-limited countries is clearly insufficient. Exacerbating the staffing shortage is a concomitant scarcity of trained laboratory technologists, histotechnologists, and cytotechnologists.

Since 2015, there has been a significant effort by national, regional and international organizations and academic institutions to increase the number of pathologists and trainees (Table 1) (8). Nigeria, Uganda, Democratic Republic of Congo, and Cuba supply pathologists in other countries with no or inadequate national staff (8). Furthermore, at least 15 countries have post-graduate trainees in other countries, mainly South Africa, Kenya, Tanzania, Uganda, Nigeria and Côte d’Ivoire.

One key component to maintaining a knowledgeable PALM workforce is access to continuing education programs (CE), which are limited in many LMICs (3, 9, 10). To address this gap, previous groups have executed successful in-person CE programs for pathologists, laboratorians, and clinicians (11–13). Although in-person activities allow for enhanced interactions between participants and trainers, they are costly to implement, challenging to scale, and may be less accessible to learners in rural areas or areas with restricted travel.

Adapting CE content to a virtual format represents a solution to these barriers (14). This need to adapt was further accelerated by limitations caused by the COVID-19 pandemic. As a small, volunteer-based organization, providing high-quality, virtual CE represented a novel strategy to synergize PO’s mission of increasing human resource capacity through engagement of our volunteer network during a time of limited international travel and collaboration with other partners active in global PALM.

In Spring 2021, we partnered with the American Society of Clinical Pathology (ASCP) to offer a free 10-week Laboratory Quality Management Systems (LQMS) course based on the World Health Organization (WHO) LQMS Essentials. An initial needs assessment included data from over 200 laboratorians representing 63 institutions, predominantly in SSA. The purpose of the needs assessment was to understand the professional background and work setting of potential participants in order to customize the content to their needs. We recruited 15 trainers from our volunteer network to virtually deliver lectures and illustrative cases to participants.

In 48 hours, 617 individuals representing 156 institutions across 24 countries registered for the course. Over 99% of registrants (n = 614) were from countries in SSA. Registrants represented all major areas of the laboratory (e.g., histology, microbiology, transfusion, chemical pathology, etc.), held a variety of positions from laboratory technician to consultant pathologist, and over 70% worked in the public sector. Live attendance at each of the 20 sessions ranged from 150 to 200 participants with many more individuals accessing the lecture recordings and materials available for free online following the course’s conclusion.

We received overwhelmingly positive feedback from the 180 participants who filled out the post-course survey. The majority of participants’ self-reported comfort and knowledge level increased in all LQMS areas following the course and over 95% were interested in taking another virtual course. Of the topics covered, participants expressed interest in receiving additional content in leadership (50.8%), process control (48.6%), and process improvement (42.9%). Moreover, several participants would have appreciated a more interactive format.

In response to this success, PO recently partnered with BIO Ventures for Global Health (BVGH) and Ahmadu Bello University Teaching Hospital (ABUTH), to launch a free 8-week virtual course on pediatric and lymph node pathology in February 2022. Representatives from PO and ABUTH developed the curriculum, recruited lecturers from our volunteer network, and moderated the virtual sessions. BVGH hosted the lectures and course content, provided administrative and technical support, and coordinated all CE credits with partnering institutions in sub-Saharan Africa. Similar to our prior experience, over 700 individuals representing 19 countries in SSA registered for this series. The average attendance at the live lectures was 219. Participants shared that they felt more confident rendering the correct diagnosis as a result of the course and that they planned to share the information they learned with their colleagues. PO is currently collaborating with ASCP and Heart to Heart International (HHI) to adapt the virtual LQMS course for a Spanish-speaking audience in Central and South America in summer 2022.

The PO experience highlights the need and enthusiasm for accessible, high-quality CE in LMICs. It also illustrates how relatively small organizations can enhance their impact on the communities they serve through utilization of a scalable platforms, partnerships with in-country institutions and like-minded organizations in HICs and leveraging a network of interested volunteers. We plan to continue offering free virtual CE as one strategy for addressing the human resource gap. However, we hope to explore creative ways of incorporating case-based interactive sessions into our virtual curriculum and to supplement our efforts with focused and strategic in-person training workshops.

### Systems

The second pillar of laboratory quality is systems. The operation and management of modern clinical laboratories is a sophisticated and complex process, the success of which requires adequate physical and technical infrastructure. One example are LISs, which play a crucial role in streamlining this workflow, reducing errors, and enabling quality indicator monitoring (15). In HICs, laboratories typically initiate the process of LIS implementation by carefully selecting a software that meets the laboratory’s unique clinical and non-clinical needs, the latter including regulatory and billing requirements and financial and infrastructure constraints. Once installed, LISs will ideally operate with minimal disruption and access to rapid resolutions, should problems arise. In HICs, this is accomplished through the availability of on-site experts trained in information technology (IT) and LIS support. Given the above considerations, the implementation and maintenance of LISs is a daunting undertaking for most laboratories in LMICs.

PO has recognized this system challenge since its inception. After forging a strategic partnership with a US-based LIS Company, Comp Pro Med, PO has successfully supported LIS implementations in partnering public laboratories in Bhutan (n = 1), Eritrea (n = 1), Ethiopia (n = 1), Malawi (n = 1) and Nigeria (n = 2). Inspired by the initial successes, Bhutan and Ethiopia have since independently expanded the system to 8 and 25 laboratories, respectively. From a sustainability perspective, all LIS implementations except one continue to operate successfully and sustainably. At least one of the laboratories has maintained their LIS for 20 years.

**FIGURE 1:**
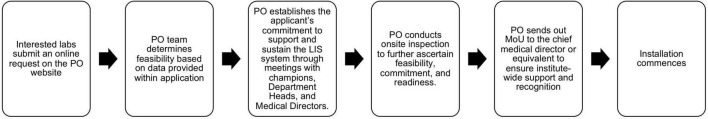
Flow chart illustrating process for vetting laboratories interested in LIS implementation.

PO continues to engage with laboratories interested in LIS implementation support through our rigorous vetting process. This process involves scrutinizing the capacity and commitment of interested laboratories, at an institutional and departmental level, to implement and independently sustain the system (Figure 1). Most recently, PO partnered with Comp Pro Med, HHI, and LIS experts from Ethiopia and Nigeria, to install an LIS at Ahmadu Bello University Teaching Hospital (ABUTH) in Nigeria. As of this writing, there are 5 pending LIS requests.

In summary, the cost of LISs can be prohibitive for many clinical laboratories with limited resources, precluding implementation of these systems despite their importance in laboratory operations. Though limited in scope, PO has adopted an approach that focuses on mentored LIS installation and maintenance in its ongoing efforts to improve clinical laboratory infrastructure in LMICs.

### Quality and accreditation

The third pillar of laboratory quality is quality and accreditation. In HICs, an essential and highly regulated component of quality and accreditation is external quality assessment (EQA), which is a system of ensuring the comparability and continued monitoring of clinical test results across different laboratories and over time (16–18). Typically, EQA providers offer performance reports that score participants’ results compared to assigned target values and results from peer groups, but there is wide variation in terminology, interpretation, and performance expectations globally (19). Laboratory professionals are often faced by the availability of numerous commercial programs, difficulty in communication with EQA providers, and lack of a sufficient number of participating peer groups for a particular measuring system (20). All of these and the additional obstacles faced by clinical laboratories in low-resource settings must be grappled with in the implementation and oversight of an EQA program in LMICs. This has led to heterogeneity in the scope and success of participation in EQA programs, and even these tend to be restricted to specific analytes or diseases (21–24).

PO has had a long and productive partnership with the Royal College of Pathologists of Australasia Quality Assurance Program (RCPAQAP) to enroll participants, free of charge, in the compact general chemistry program. This program consists of 6 surveys mailed at the beginning of the year with set dates for result submission for each survey over the subsequent months. Each survey includes two levels of commonly ordered tests which are scored on comparability to target and peer-group results. Since 2009, PO has enrolled up to 24 laboratories from 6 countries in this program with the majority of labs participating for at least three years and one lab continuing to participate since enrollment in 2014. Between 2009 and 2014, PO capitalized on the effectiveness of in-country volunteer efforts and the simpler-to-implement short-term educational schemes to track EQA performance before and after a 12–18-month period of active feedback on survey reports. This entailed site visits to each laboratory for initial assessment and training, continued follow-up by a PO volunteer during the active feedback phase, and the securing of external funding, which have proved to be limiting factors long-term.

Since 2019 to the present day, PO and RCPA continue to support 6 laboratories, with plans to enroll more in the coming 2 years. Despite challenges with inconsistent timely and complete result submission, often due to inconsistent access to reagents, supplies, service, and dedicated personnel and, more recently, the COVID-19 pandemic, a pattern of steadily improving EQA performance has been observed in this cohort.

Our current goal is to shift emphasis from costly and laborious on-site EQA training to virtual approaches that can reach out to a wider audience. Annual training workshops in one regionally accessible location may then supplement these courses. In this as in other endeavors, the support of volunteers from our network who are willing to engage with laboratories on an individual basis or small-group basis remains crucial for sustainable progress.

## Discussion

It is undeniable that PALM services are critical to functional health systems globally; however, building sustainable capacity for these services in LMICs continues to be a challenge. One potential barrier to capacity building efforts is the fragmented and disease-specific nature of many global health programs. PO demonstrates that a network of dedicated volunteers, in partnership with corporations and professional and non-profit organizations, can initiate sustainable change in the quality of PALM services in LMICs by focusing their combined efforts on the three pillars of laboratory quality: human resources, systems, and quality and accreditation.

## Data availability statement

The original contributions presented in this study are included in the article/supplementary material, further inquiries can be directed to the corresponding author.

## Author contributions

AN collected the information featured in Table 1. All authors contributed to the conception, drafted components, and approve of the final version of the work submitted.
